# Association of early childhood lifestyle patterns with overweight in preschoolers and timing of adiposity rebound

**DOI:** 10.1186/s12966-026-01941-w

**Published:** 2026-06-25

**Authors:** Mélissa Baietto, Camille Le Gal, Alexandra Descarpentrie, Marion Lecorguillé, Aminata H. Cissé, Jonathan Y. Bernard, Muriel Tafflet, Jade Pilato, Judith van der Waerden, Blandine de Lauzon-Guillain, Marie-Aline Charles, Barbara Heude, Sandrine Lioret

**Affiliations:** 1 Université Paris Cité and Université Sorbonne Paris Nord, Inserm, INRAE, Centre for Research in Epidemiology and Statistics (CRESS), Paris, 75004 France; 2 Department of Pediatrics, The Saban Research Institute, Children’s Hospital Los Angeles, University of Southern California, Los Angeles, CA USA; 3 University of Exeter, Clinical and Biomedical Sciences, Exeter, EX1 2LU UK; 4 Université de Montpellier, Inserm, Desbrest Institute of Epidemiology and Public Health, Inserm, Montpellier, 34090 France; 5 Social Epidemiology, Mental Health and Addictions Team (ESSMA), Pierre Louis Institute of Epidemiology and Public Health, INSERM, Sorbonne University, Paris, 75012 France; 6 Ined, Inserm, EFS, joint unit Elfe, Aubervilliers, France

**Keywords:** Childhood obesity, Adiposity rebound, Breastfeeding, Infant nutrition, Diet, Physical activity, Sedentary behavior, Television, Sleep, Birth cohort

## Abstract

**Background:**

An unhealthy diet, excessive screen time, low levels of physical activity and suboptimal sleep are known risk factors for childhood overweight and obesity (OW/OB). These energy balance–related behaviors tend to co-occur and combine into “lifestyle patterns” (LPs) that may have a synergistic effect on OW/OB. However, few studies have examined these LPs before preschool age, and none investigated the possible involvement of parental feeding practices (e.g., breastfeeding and timing of complementary feeding), non-screen leisure activities and sleep quality. We aimed to identify such LPs during the first 2 years of life and assess their associations with OW/OB at age 5 years and age at adiposity rebound (AR).

**Methods:**

Participants were children from the French nationwide ELFE (*Etude Longitudinale Française depuis l’Enfance*) birth cohort. We used principal component analysis of 18 items characterizing parental feeding practices and children’s energy balance-related behaviors (*n* = 13,121) to derive LPs. We analyzed the associations of LPs with OW/OB at age 5 years (International Obesity Task Force definition; *n* = 8,388) and age at AR (in days; *n* = 7,845) using multivariable logistic and linear regression models, respectively.

**Results:**

We identified three LPs: “Early complementary feeding, discretionary consumption, high screen time”, “Balanced diet, non-screen leisure activities”, and “Healthy feeding practices, low dairy consumption, suboptimal sleep” patterns. The first pattern was positively associated with OW/OB at age 5 years (OR [95% CI] = 1.09 [1.03, 1.16] per 1-SD increase) and inversely associated with age at AR (β [95% CI] = − 12.1 days [− 19.1; −5.0] per 1-SD increase). No association was observed for the second LP. For the third pattern, no clear evidence of associations was found, although the effect sizes were comparable to those of the first LP (OR = 1.07 [0.99; 1.15]; β = −7.6 days [− 15.5; 0.3] per 1-SD increase).

**Conclusion:**

These results emphasize the perinatal period as a critical window for the emergence of interrelated lifestyle behaviors associated with OW/OB. They highlight the need for interventional studies to evaluate the effectiveness of early, integrated, multi-behavioral strategies to prevent childhood obesity.

**Supplementary Information:**

The online version contains supplementary material available at https://doi.org/10.1186/s12966-026-01941-w.

## Background

In 2024, approximately 35 million children worldwide who were < 5 years old were living with overweight or obesity (OW/OB) [1]. This situation is a major public health concern because early childhood OW/OB substantially increases the risk of OW/OB in adulthood [2] and is associated with multiple physical and mental comorbidities, including type 2 diabetes mellitus, hypertension, and depression [3].

Within the framework of the Developmental Origins of Health and Disease theory (DOHaD) [4], some risk factors for childhood OW/OB are already in play during the “first 1,000 days” of life (i.e., from conception to age 2 years) [5]. In the prenatal period, these risk factors include pre-pregnancy parental OW/OB, smoking during pregnancy, and high birth weight [6–8]. Although these prenatal factors shape early susceptibility to OW/OB, postnatal behaviors represent additional influences on childhood weight outcomes. In particular, parental feeding practices, such as no or short breastfeeding and the introduction of complementary feeding before age 4 months, have also been associated with childhood OW/OB [8–10]. Moreover, energy-dense, ultra-processed, and nutrient-poor diets; excessive screen time; low physical activity; and short sleep duration have all been linked to increased risk of childhood OW/OB [8, 11–13]. Several studies have further demonstrated that these energy balance-related behaviors (EBRBs) tend to co-vary and combine into so-called lifestyle patterns (LPs), which may have a synergistic effect on risk of childhood OW/OB [14–16]. However, only three previous studies have examined LPs during the first 2 years of life [17–19], and to our knowledge, none included parental feeding practices or child non-screen leisure activities or sleep quality in their construction.

Additionally, during early childhood, body mass index (BMI) follows a characteristic trajectory: after a rapid increase in BMI during infancy, BMI decreases to a nadir at about age 6 years before a second increase, called adiposity rebound (AR), which lasts until the end of growth [20]. Early age at AR (before age 5 years) is an established risk factor for OW/OB in adolescence and adulthood [6, 21]. Therefore, age at AR provides a valuable complementary outcome for studying prospective associations between early determinants and childhood OW/OB.

In this context, our study aimed to (1) identify children’s LPs based on parental feeding practices and child diet, screen time, and other leisure activities as well as sleep during the first 2 years of life; and (2) examine the associations of these LPs with OW/OB at age 5 years and age at AR.

## Methods

### Study design and participants

The French ELFE (*Etude Longitudinale Française depuis l’Enfance*) study is a nationwide population-based birth cohort [22]. In 2011, it enrolled 18,329 children born in a randomly selected sample of 344 maternity units in mainland France over 25 days distributed across four periods, representing the four seasons. Inclusion criteria were single or twin births at ≥ 33 weeks of gestation and mothers aged ≥ 18 years who did not plan to leave mainland France within 3 years. To facilitate the inclusion of women with limited French literacy, the information letter and consent form were translated into Arabic, Turkish, and English, the three most commonly spoken languages among non-French speakers in France. Among eligible mothers, 51% agreed to participate and provided written informed consent for themselves and their child. When present at inclusion, fathers also signed consent for their child’s participation or were informed of their right to oppose it.

### Measurements

#### Parental feeding practices and EBRBs in the first 2 years of life

Information on parental feeding practices was collected monthly between age 2 and 10 months via parental questionnaires, and children’s EBRBs were assessed at age 2 years. These data were used to derive patterns integrating parental feeding practices and EBRBs, hereafter called LPs for conciseness.

##### Parental feeding practices

 Indicators of parental feeding practices during the first months of life were breastfeeding duration (in months), age at introduction of complementary feeding (before age 4 months, yes/no), and age at first introduction of sweet beverages (fruit juice or sugar-sweetened beverages; before age 10 months, yes/no).

##### Diet

 At age 2 years, data from a Food Frequency Questionnaire were used to determine the frequency of consumption for 20 dietary items [23]. In this study, the food classification was established based on similarities in food type, energy density, and context of consumption, consistent with previous studies [15, 16]. Of the 14 food items selected, 10 were combined into the following groups: “vegetables” (raw and cooked), “fruits” (fresh and stewed), “discretionary savory food” (chips or salty biscuits, and quiches, pizzas or savory pies), “discretionary sweet food” (Viennese pastries, cookies or cakes, and chocolate or sweets), “sweet beverages” (fruit juice and sugar-sweetened beverages). Discretionary foods and beverages were defined as items characterized by high energy density and low micronutrient content, consistent with previous literature [15, 17, 24]. The original food groups “fish”, “dairy products” (yogurts and *“petits suisses”*), “processed meat” (which in the French context typically includes products such as sausages, pâté, ham, and cured meats) and “French fries” items were retained as such. The seven possible responses, ranging from “Never” to “Several times a day”, were converted into monthly frequencies (“Never” was coded as 0; “Less often” as 1 time per month; “Several times a month” as 3 times per month; “Several times a week” as 12 times per month; “Once a day” as 30 times per month; and “several times a day” as 60 times per month) and summed when food/beverage groups included several items.

##### Screen time

 Screen time at age 2 years (in hours/day) was calculated as the average over a full week, weighting the responses for both weekdays and weekends. It reflected the total duration of screen use across various devices, including television, tablets, computers, video games, consoles, and smartphones.

##### Non-screen leisure activities

 Participation in non-screen leisure activities at age 2 years was assessed and grouped into three categories [25, 26]: educational games (drawing or painting, stacking toys, nesting toys, puzzles), pretend play (with stuffed animals, dolls or “*poupons*”, and toy cars), and movement activities (playing with a ball, walking with parents). Pretend play activities were grouped together to limit the influence of sex differences associated with gender-oriented play. The four response options, ranging from “Never” to “Every day”, were converted into weekly frequencies (“Never” was coded as 0; “Occasionally” as 1 time per week; “Often but not every day” as 4 times per week; “Every day” as 7 times per week) and summed within each of the three categories.

##### Sleep

 Total sleep duration at age 2 years (in hours/day) included both night-time sleep and daytime naps and was calculated as the average over a full week, weighting the responses for weekdays and weekends. Additionally, sleep quality was assessed by summing the weekly frequencies of two indicators: difficulty falling asleep (“Never” was coded as 0; “Sometimes” as 1.5 times per week; “Often” as 3.5 times per week) and night-time waking (“Never” was coded as 0; “1 to 2 nights per week” as 1.5 times per week, “≥ 2 nights per week” as 3.5 times per week).

#### Outcomes

Anthropometric measures were collected by telephone interview when the child was approximately 5 years old. Parents were asked to report their child’s most recent weight and height as recorded by health professionals in the child health booklet. Child BMI was calculated as weight (kg)/(height [m])². We used the International Obesity Task Force age-and sex-specific references and cut-offs to assess OW/OB versus thin/normal BMI [27]. The age at AR was estimated as a continuous variable (in days) by using growth modelling based on repeated anthropometric measurements to model individual BMI trajectories with cubic mixed-effects models, as described in detail elsewhere [28].

#### Covariates

We considered variables that have been found to be associated with OW/OB, age at AR and at least one children’s EBRB [7, 8, 28, 29]. Maternal age at delivery, parity (primiparous versus multiparous), pre-pregnancy maternal BMI (underweight, normal weight, overweight, obesity) and maternal smoking during pregnancy (yes/no), were collected through in-person interviews at the maternity hospital upon delivery. Cesarean delivery (yes/no), child’s sex, and birth weight (continuous) were obtained from medical records. Parents’ migration status (non-immigrant, at least one parent descendant of immigrant, at least one parent immigrant); parents’ education level, based on the highest level attained by either parent (≥ 3-year university, 1- to 2-year university, ≤ high school degree); household income (in quintiles); and paternal BMI (continuous) and smoking status (yes/no) were collected by computer-assisted telephone interview when the child was 2 months old.

### Study population

**Fig. 1:**
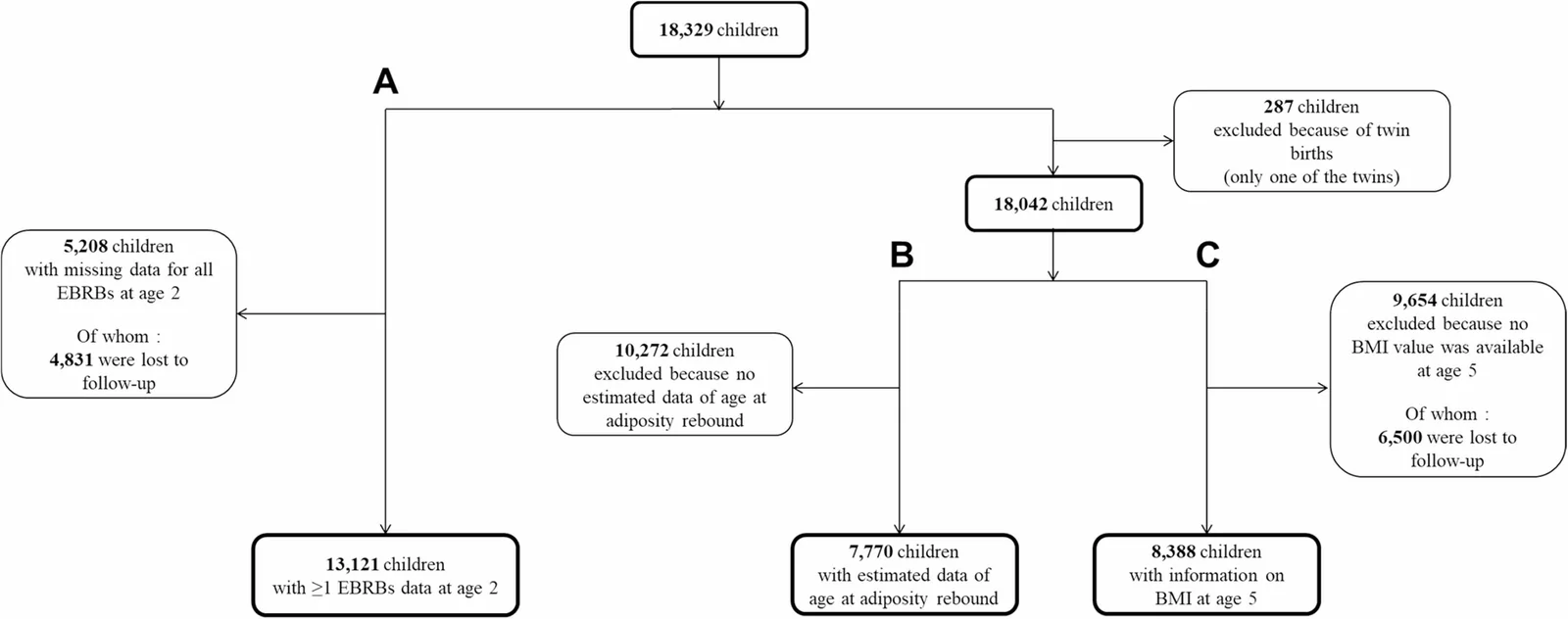
Flow chart of the study sample. **A **lifestyle patterns analysis, **B** overweight/obesity analysis, **C** adiposity rebound analysis. EBRB: energy balance-related behavior; BMI: body mass index

For the derivation of LPs, we analyzed data for all children, including twins, with at least one available EBRB measure at age 2 years (*n* = 13,121). For all multivariable analyses, we started with the full cohort of children (*n* = 18,329) and randomly selected one child from each twin pair (*n* = 287). For the analysis of OW/OB, we included children with a BMI measurement from age 4.1 to 6.5 years (mean: 5.0 years; *n* = 8,388) and for the AR analysis, those with an estimated age at AR (*n* = 7,770) **(**Fig. 1**)**.

### Statistical analysis

We compared sociodemographic factors of participants included in and excluded from the analysis by Wilcoxon rank sum test for numerical data and chi-squared test for categorical data.

Principal component analysis (PCA) is a global and an integrative method used to synthesize the information contained in a large number of correlated variables into a smaller number of independent dimensions (or “components”) [30]. We used this method to derive LPs from the three parental feeding practices and 15 EBRBs items (*FactoMineR* R package). The correlation matrix of these variables is provided in Supplementary Table 1 for further details. Missing data ranged from 0% for the three categories of non-screen leisure activities to 26.5% for age at first introduction of sweet beverages (Supplementary Table 2) and were imputed using the regularized iterative PCA method (*missMDA* R package) [31]. The number of patterns retained was determined based on the scree plot and the eigenvalue ≥ 1 criterion [32]. To interpret each pattern, we considered the items most strongly related to it, that is, those with an absolute loading coefficient (the correlation between each standardized variable and the given LP) > 0.30 [30]. An individual score for each LP, standardized by construction, was calculated by summing the standardized values of each variable weighted by the PCA loadings. A score of 0 represents the average level of adherence to the pattern across the analytical sample. A score of + 1-SD indicates a child whose combination of parental feeding practices and EBRBs more strongly aligns with the identified pattern, reflecting a shift from average adherence to high adherence. However, the term “adherence” should be interpreted with caution, as it does not necessarily imply deliberate choice, despite its connotation.

We applied the Multivariate Imputation by Chained Equations package in R to impute missing LPs scores and covariates, including all relevant variables informing the imputation process, under the missing at random assumption (*mice* R package) [33]. Imputations were conducted separately for the analysis of OW/OB at age 5 years (Supplementary Table 3) and age at AR (Supplementary Table 4). For each analysis (OW/OB and age at AR), we generated 50 separate imputed datasets. We conducted plausibility checks by comparing the distributions of observed and imputed values, which were similar, and by verifying that imputed values fell within realistic ranges. In addition, convergence of the imputation algorithm was assessed by inspecting the stability of parameter estimates across iterations, which did not show evidence of systematic drift, indicating satisfactory convergence. The results of the imputed datasets were combined with the MIcombine function in R, which estimates combined effects and their 95% confidence intervals, aligning with Rubin’s rules [34].

We performed multivariable logistic regression to assess associations between LPs and OW/OB at age 5 years and multivariable linear regression to estimate associations between LPs and age at AR. These models included all LPs (independent because orthogonal by construction) and were adjusted for the covariates described above.

Sensitivity analyses were conducted with complete cases for the LPs (*n* = 8,675), OW/OB (*n* = 6,224), and AR (*n* = 6,230) analyses.

All analyses were conducted using RStudio (2022) (RStudio: Integrated Development Environment for R. RStudio, PBC, Boston, MA).

## Results

### Participant characteristics

As compared with children excluded from the LPs analysis, those included were more frequently born to older or non-single mothers and to parents without a migration background, with higher educational attainment (undergraduate degree or above), or with higher household income (Table 1). For the anthropometrics analyses, in addition to these differences, included children were more frequently born to primiparous mothers (Supplementary Tables 5–6). Among the 8,388 children with available BMI data at age 5 years, 7.9% were classified as OW/OB. Among the 7,845 children with available data on age at AR, the mean (SD) age at AR was 1,921 (399) days (or 5.26 [1.09] years).

**Table 1:** Characteristics of parents and children in the lifestyle patterns analysis. The ELFE study (*n* = 18,329)

	Included *N* = 13,121	Excluded *N* = 5,208	*p*-value^a^
Maternal age at delivery^b^ (years)	30.9 (27.9–34.3)	29.0 (25.2–33.2)	< 0.001
Single motherhood			< 0.001
Yes	2.7% (345)	9.3% (338)	
Parity (first child)			0.842
Yes	45.9% (5,941)	46.1% (2,307)	
Parents’ migration status			< 0.001
Non-immigrant	70.2% (8,120)	51.1% (1,080)	
At least one parent descendant of immigrant	17.5% (2,025)	18.5% (391)	
At least one parent immigrant	12.3% (1,419)	30.4% (643)	
Parents’ education level			< 0.001
≥ 3-year university	20.7% (6,516)	24.6% (879)	
1- to 2-year university	22.2% (2,861)	17.3% (616)	
≤ High school degree	27.1% (3,482)	58.1% (2,071)	
Household income per consumption unit^b^ (€/month)	1,571 (1,190–1,958)	1,222 (833–1,667)	< 0.001
Sex of the child			0.007
Girl	49.2% (6,459)	47.0% (2,400)	

^a^ Wilcoxon rank sum test for numeric data; chi-squared test for categorical data

^b^ Median (IQR)

### Lifestyle patterns

We identified three LPs, explaining 32% of the total variance **(**Table 2**)**. The first pattern, labelled “Early complementary feeding, discretionary consumption, high screen time”, was characterized by early introduction of complementary foods and sweet beverages, along with a high frequency of energy-dense, nutrient-poor food consumption at age 2 years, as well as high levels of screen time. The second pattern, “Balanced diet, non-screen leisure activities”, was defined by frequent consumption of fruits, vegetables, and fish, along with regular participation in leisure activities other than screen viewing. The third pattern, “Healthy feeding practices, low dairy consumption, suboptimal sleep”, was characterized by a long duration of breastfeeding, introduction of complementary foods after age 4 months, low dairy consumption frequency at age 2 years, and short sleep duration and poor sleep quality at the same age.

**Table 2:** Analysis of behavioral variables and principal component analysis loadings for lifestyle patterns (LPs). The ELFE study (*N* = 13,121)

	Median (IQR)^a^	PCA Loadings		
		LP1^b^	LP2^c^	LP3^d^
Breastfeeding duration (months)	2.0 (0.0–5.8)	-0.13	0.09	**0.59**
Introduction of complementary feeding before age 4 months (yes)	19.7%	**0.47**	-0.09	**-0.32**
Introduction of sweet beverages before age 10 months (yes)	40.2%	**0.55**	-0.04	-0.08
Vegetables (/day)	1.1 (0.9–2.0)	-0.17	**0.63**	0.20
Fruits (/day)	1.4 (0.9–2.4)	-0.02	**0.62**	0.15
Fish (/day)	0.4 (0.1–0.4)	0.15	**0.42**	0.10
Dairy products (/day)	2.0 (1.0–2.0)	0.06	0.27	**-0.35**
Discretionary savory foods (/day)	0.1 (0.1–0.2)	**0.62**	0.07	0.15
Discretionary sweet foods (/day)	1.0 (0.5–1.4)	**0.55**	0.06	0.03
Processed meat (/day)	0.0 (0.0–0.1)	**0.47**	0.09	0.08
French fries (/day)	0.1 (0.0–0.1)	**0.64**	0.07	0.10
Sweet beverages (/day)	0.4 (0.1–1.0)	**0.63**	0.07	0.06
Educational games (/week)	16.0 (11.0–19.0)	-0.13	**0.55**	-0.09
Pretend play (/week)	12.0 (7.0–16.0)	-0.01	**0.45**	-0.16
Movement activities (/week)	10.0 (7.0–12.0)	0.16	**0.47**	-0.09
Total screen time (hours/day)	0.6 (0.2–1.1)	**0.45**	-0.14	-0.07
Total sleep duration (hours/day)	13.0 (12.4–13.6)	-0.03	0.12	**-0.57**
Trouble falling asleep & night-time awakenings (/week)	1.5 (0.0–3.5)	0.16	-0.11	**0.54**
% variance explained		14.4	10.2	7.6

^a^ Values are % yes or median (IQR); ^b^ LP1: “Early complementary feeding, discretionary consumption, high screen time”; ^c^ LP2: “Balanced diet, non-screen leisure activities”; ^d^ LP3: “Healthy feeding practices, low dairy consumption, suboptimal sleep”. In bold, absolute value of factor loadings ≥ 0.30

### Multivariable analysis

The “Early complementary feeding, discretionary consumption, high screen time” LP was positively associated with OW/OB at age 5 years (odds ratio [OR] [95% CI] = 1.09 [1.03, 1.16] per 1-SD increase) **(**Table 3**)** and inversely associated with age at AR (β [95% CI] = − 12.0 days [− 19.2; −4.9] per 1-SD increase).

The “Healthy feeding practices, low dairy consumption, suboptimal sleep” pattern showed imprecise estimates of a similar magnitude to the first pattern, though confidence intervals included the null, indicating no clear evidence of an association with both OW/OB at age 5 (OR = 1.07 [0.99; 1.15]) and age at AR (β = −7.6 days [− 15.5; 0.3] per 1-SD increase). We found no associations between the “Balanced diet, non-screen leisure activities” pattern and anthropometric outcomes.

**Table 3:** Associations between lifestyle patterns (LPs) and anthropometric outcomes*. The ELFE study

	Overweight/obesity at age 5 *N* = 8,388		Age at adiposity rebound *N* = 7,770	
	OR [95% CI]	*p*-value	β [95% CI]	*p*-value
LP1^a^	1.09 [1.03, 1.16]	0.003	-12.0 [-19.2, -4.9]	< 0.001
LP2^b^	0.98 [0.92, 1.04]	0.511	0.9 [-5.9, 7.7]	0.789
LP3^c^	1.07 [0.99, 1.15]	0.082	-7.6 [-15.5, 0.3]	0.060

*Logistic regression model for overweight/obesity and linear regression model for age at adiposity rebound. These two multivariable models included the three LPs and were adjusted for child sex, maternal age at delivery, parity, maternal and paternal BMI, maternal smoking during pregnancy, paternal smoking, cesarean delivery, birth weight, household income, parents’ migration status and parents’ education level

^a^ LP1: “Early complementary feeding, discretionary consumption, high screen time”; ^b^ LP2: “Balanced diet, non-screen leisure activities”; ^c^ LP3: “Healthy feeding practices, low dairy consumption, suboptimal sleep”

### Sensitivity analyses

First, the LP analysis restricted to complete cases yielded consistent patterns, and second, on multivariable analyses, the effect sizes were comparable across the two anthropometric outcomes (Supplementary Tables 7–8).

## Discussion

This study extends the literature on LPs by focusing on the first 2 years of life and integrating parental feeding practices, sleep, and non-screen leisure activities. We identified three distinct LPs: “Early complementary feeding, discretionary consumption, high screen time”, “Balanced diet, non-screen leisure activities”, and “Healthy feeding practices, low dairy consumption, suboptimal sleep”. The first LP was positively associated with childhood OW/OB at age 5 and inversely associated with age at AR. No association was observed for the second LP. For the third LP, no clear evidence of associations was found, although the effect sizes were comparable to those of the first LP.

### Lifestyle patterns identification

On the whole, the three LPs explained about one third of the total variance, which is consistent with the range reported in similar studies on LPs in children [17, 19, 24].

The combination within a single pattern of discretionary food and beverage consumption and increased screen time has been consistently documented across countries, especially among school-aged children, less frequently among preschoolers and only rarely before age 3 years [14–19]. Several mechanisms have been proposed. Television viewing or movie watching, as largely passive hands-free activities, may facilitate snacking; exposure to junk food and fast-food advertising may increase children’s requests for these foods; and eating while watching screens may distract from internal satiety cues, potentially leading to overconsumption [18, 35, 36]. These mechanisms should, however, be interpreted cautiously, as the observed patterns reflect statistical covariance structures rather than causal or unified behavioral processes. In our study, this pattern was characterized by additional, earlier features, notably the introduction of complementary feeding before age 4 months and sweet beverages before 10 months. This observation is consistent with previous findings from the ELFE study linking added sugar intake in infancy to early complementary feeding [37] and with evidence of strong tracking of discretionary food and beverage consumption from early childhood onward reported in other studies [38, 39].

The “Balanced diet, non-screen leisure activities” pattern identified resembles the “healthy” LPs frequently described in school-aged children, which are typically characterized by a nutrient-rich diet combined with high levels of physical activity, low screen time, and/or, although less often examined, adequate sleep [15–17]. However, in children ≤ 2 years old, physical activity mainly takes the form of spontaneous bursts of active play rather than sustained or structured exercise. Therefore, the novelty of our approach was to include active play, educational games, and pretend play as proxies for leisure activities that encourage movement, foster motor skills, stimulate imagination, and promote creativity. These findings show that early participation in a variety of non-screen activities and good diet quality at age 2 years tended to co-occur. Although total screen time and total sleep duration contributed less strongly to this pattern, their factor loadings were nevertheless in the expected direction.

To the best of our knowledge, the “Healthy feeding practices, low dairy consumption, suboptimal sleep” pattern, characterized by prolonged breastfeeding, complementary feeding after age 4 months, and short, poor-quality sleep at age 2 years, has not previously been reported in the literature. Previous results from the ELFE cohort showed that prolonged breastfeeding was associated with complementary feeding after age 4 months [40] and with short and poor-quality sleep at 1 year [41], suggesting that parental practices related to breastfeeding, such as bed sharing, nursing to sleep or at night, and parental presence at bedtime, may partly explain this latter association [42, 43]. Consistent with the only study that examined sleep in LP analyses at age 2 years, sleep did not covary with diet, screen time, or physical activity [18], likely reflecting the wide variability in sleep among young children, which may not directly depend on other EBRBs [44]. Although sleep quality has rarely been included in LP analyses [16], several studies have reported negative associations between sleep duration and night time waking at age 2 years [45, 46].

### Lifestyle patterns and anthropometric outcomes

The “Early complementary feeding, discretionary consumption, high screen time” pattern was positively associated with OW/OB at age 5 years. This finding was expected, as each defining behavior has been individually linked to OW/OB [8, 9, 11, 12]. While similar LPs, characterized by high consumption of discretionary foods and drinks and increased screen time, have been associated with OW/OB in school-aged children [14–16], our findings further highlight that these influences may emerge earlier in life, in combination with parental feeding practices, consistent with the DOHaD paradigm. Using age of AR as a complementary outcome, we also observed an inverse association, strengthening the robustness of our findings. This is noteworthy given that earlier AR has been associated with less favorable outcomes later in childhood [29]. Together, these results suggest that early-life practices and behaviors, individually and in combination, may have a cumulative and/or synergistic effect on risk of OW/OB, potentially through early programming of energy metabolism and appetite regulation [4]. Although causality cannot be inferred, the prospective design, temporal ordering of exposures and outcomes, and consistency with prior evidence support its plausibility.

In contrast, the “Balanced diet, non-screen leisure activities” pattern showed no association with OW/OB. This finding may reflect that the dietary items characterizing this pattern were predominantly micronutrient-rich and energy-poor but also that the specific contribution of non-screen leisure activities assessed at age 2 years to overall energy expenditure remains uncertain. Nevertheless, the positive influence of these activities on motor skills (shown to predict increased levels of physical activity later in childhood [47]) warrants consideration. Moreover, healthy LPs tend to track less consistently from early childhood than unhealthy ones [17, 48, 49], potentially resulting in less sustained exposure and thereby attenuating their observable protective effect by age 5.

Finally, while we found no clear evidence of associations for the “Healthy feeding practices, low dairy consumption, suboptimal sleep” pattern, the point estimates were notably similar to those of the first pattern. Being mixed by construction, this result is difficult to interpret. Short and poor-quality sleep are generally associated with increased OW/OB risk [12], although studies examining sleep and age at AR are scarce. In contrast, longer breastfeeding and later complementary feeding are generally linked to reduced risk of OW/OB (although the effect of breastfeeding is only moderate [10] and was not observed in ELFE [50]) and to later age at AR [9, 29, 51]. Thus, if an association exists, suboptimal sleep may pull the association toward increased risk of OW/OB, and healthy parental feeding practices may attenuate it.

### Implications for childhood obesity prevention and health promotion

Considering EBRBs and parental feeding practices as patterns rather than isolated behaviors provides a more comprehensive and integrated view of many correlated variables, better reflecting real life situations. Because these interconnected behaviors have each been linked to OW/OB, examining them together provides deeper insight into how they combine and are associated with OW/OB and age at AR. Although the effect sizes observed may appear modest at the individual level, a 1-SD increase in adherence to the “Early complementary feeding, discretionary consumption, high screen time” pattern was associated with a 9% increase in the odds of OW/OB, while a contrast between low (− 2 SD) and high (+ 2 SD) adherence corresponds to an approximately 41% increase in odds. Given that these patterns represent common, modifiable exposures that emerge early in life, track across childhood, and are therefore amenable to prevention [17, 48, 49], their public health relevance may be substantial. Furthermore, given the multifactorial and cumulative nature of obesity, even modest effects at the individual level may have meaningful implications at the population level. The resulting patterns highlight the value of multi-behavioral interventions, which are generally more effective than those targeting a single behavior [52, 53]. The absence of a significant association between the second LP, “Balanced diet, non-screen leisure activities”, and OW/OB does not imply that this pattern is unimportant. Any potential protective effect on BMI might emerge later in childhood, especially after AR, which could partly obscure associations at age 5, though this hypothesis remains to be investigated in longitudinal studies. However, beyond weight, this pattern may still benefit other aspects of health, as non-screen leisure activities are key to cognitive, social, emotional, and physical development [54]. Therefore, early-life interventions should prioritize support for appropriate complementary feeding, along with efforts to reduce television time and promote creative and movement-based leisure activities, as well as healthy eating and optimal sleep habits and routines [55, 56]. Such integrated interventions may provide synergistic benefits by influencing health behaviors simultaneously, beyond the simple additive effects of targeting individual behaviors in isolation, and may hold promise for fostering healthier multi-behavioral trajectories from the very start of life.

### Strengths and limitations

This study has several limitations. First, parental self-reports are subject to measurement error, including social desirability bias and limited precision, as compared with objective methods such as accelerometry for physical activity [57]. However, although accelerometry provides objective information on activity duration and intensity, the measures we used offer complementary insights into types and contexts of activities, which are particularly relevant for informing obesity prevention interventions. Regarding anthropometric data, although weight and height were reported by parents, they were extracted from measurements recorded by health professionals in the child health booklet, which likely limits systematic reporting bias. Nevertheless, some degree of measurement imprecision may persist, which is expected to be non-differential with respect to exposures and therefore likely to attenuate the observed associations rather than introduce spurious ones. Moreover, BMI cannot distinguish between fat mass and lean mass, a limitation that may be particularly relevant for LPs featuring physical activity. In addition, selection and attrition biases have led to an over-representation of families with higher socioeconomic position, which limits the generalizability of our findings to more disadvantaged populations and may have reduced the variability in lifestyle exposures, potentially attenuating the magnitude of the estimated associations. Additionally, although our primary analyses rely on the missing at random assumption, complete case sensitivity analyses yielded consistent LPs and effect estimates. This consistency suggests that potential violations of missing at random assumption are unlikely to have meaningfully biased our results. However, we cannot exclude the possibility of residual bias due to unmeasured factors related to both attrition and confounding, including family or environmental characteristics (e.g., parenting styles, childcare context, neighborhood characteristics, or genetic predisposition to obesity).

This study also has strengths. First, the large sample size, prospective design, and adjustment for multiple confounders strengthen the robustness of the analyses. Another strength is the focus on the first 2 years of life, a period that remains largely understudied in the LP literature. Additionally, including parental feeding practices, non-screen leisure activities, and sleep quality in the pattern analysis, along with dietary intake, screen time, and sleep duration, provided novel insights into correlated behaviors in young children [16]. Finally, the use of two approaches to assess OW/OB risk, namely BMI and age at AR, further strengthens the robustness of the study.

## Conclusions

In the ELFE study, we identified three LPs: “Early complementary feeding, discretionary consumption, high screen time”, “Balanced diet, non-screen leisure activities”, and “Healthy feeding practices, low dairy consumption, suboptimal sleep”. The first pattern was positively associated with childhood OW/OB at age 5 and inversely associated with age at AR. For the third pattern, no clear evidence of associations was found, although the effect sizes were comparable to those of the first LP. These findings emphasize the critical role of the perinatal and early postnatal periods in the development of early, interrelated feeding practices and lifestyle behaviors associated with OW/OB risk. They underscore the need for interventional studies to evaluate the effectiveness of integrated, early-life, multi-behavioral strategies to prevent childhood OW/OB.

## Supplementary Information

Supplementary Material 1.

Supplementary Material 2.

## Data Availability

The datasets analyzed during the current study is not publicly available due to ethical restrictions related to protection of participant confidentiality and legal restrictions imposed by the French National Commission on Data Processing and Liberties (CNIL). Investigators who wish to access the data reported in this article must address a reasonable request to the Elfe’s data access committee (CADE) at contact@elfe-france.fr.

## Abbreviations

AR: Adiposity rebound
BMI: Body mass index
DOHaD: Developmental origins of health and disease theory
EBRB: Energy balance-related behavior
LP: Lifestyle pattern
OW/OB: Overweight or obesity
PCA: Principal component analysis
