# Supplementary material for: Association of early childhood lifestyle patterns with overweight in preschoolers and timing of adiposity rebound
Source: Int J Behav Nutr Phys Act. 2026 Jun 25;23:86. doi: 10.1186/s12966-026-01941-w (PMC13587429; doi:10.1186/s12966-026-01941-w)
Supplement: Supplementary file 1 [file 12966_2026_1941_MOESM1_ESM.docx]

**Supplementary material**

***Supplementary Table 1.*** *Matrix of correlations between parental feeding practices and energy balance-related behaviors*. The ELFE study with complete cases (N=8,675).*

| **Variables** | Breastfeeding duration | Introduction of complementary feeding before age 4 months | Introduction of sweet beverages before age 10 months | Vegetables | Fruits | Fish | Dairy products | Discretionary savory foods | Discretionary sweet foods | Processed meat | French fries | Sweet beverages | Educational games | Role-playing games | Moving activities | Total screen time | Total sleep duration | Trouble falling asleep & night-time awakenings |
| --- | --- | --- | --- | --- | --- | --- | --- | --- | --- | --- | --- | --- | --- | --- | --- | --- | --- | --- |
| Breastfeeding duration | 1.00 |  |  |  |  |  |  |  |  |  |  |  |  |  |  |  |  |  |
| Introduction of complementary feeding before age 4 months | -0.14 | 1.00 |  |  |  |  |  |  |  |  |  |  |  |  |  |  |  |  |
| Introduction of sweet beverages before age 10 months | -0.03 | 0.27 | 1.00 |  |  |  |  |  |  |  |  |  |  |  |  |  |  |  |
| Vegetables | 0.12 | -0.09 | -0.06 | 1.00 |  |  |  |  |  |  |  |  |  |  |  |  |  |  |
| Fruits | 0.07 | -0.06 | -0.01 | 0.34 | 1.00 |  |  |  |  |  |  |  |  |  |  |  |  |  |
| Fish | -0.03 | 0.01 | 0.02 | 0.16 | 0.13 | 1.00 |  |  |  |  |  |  |  |  |  |  |  |  |
| Dairy products | -0.13 | 0.02 | -0.02 | 0.06 | 0.09 | 0.04 | 1.00 |  |  |  |  |  |  |  |  |  |  |  |
| Discretionary savory foods | -0.01 | 0.08 | 0.09 | -0.04 | 0.01 | 0.08 | 0.02 | 1.00 |  |  |  |  |  |  |  |  |  |  |
| Discretionary sweet foods | -0.04 | 0.08 | 0.09 | -0.04 | 0.00 | 0.02 | 0.09 | 0.24 | 1.00 |  |  |  |  |  |  |  |  |  |
| Processed meat | -0.02 | 0.08 | 0.08 | -0.01 | -0.01 | 0.02 | 0.01 | 0.21 | 0.16 | 1.00 |  |  |  |  |  |  |  |  |
| French fries | -0.03 | 0.09 | 0.11 | -0.07 | 0.02 | 0.10 | 0.02 | 0.38 | 0.25 | 0.21 | 1.00 |  |  |  |  |  |  |  |
| Sweet beverages | 0.00 | 0.13 | 0.27 | -0.03 | 0.06 | 0.03 | 0.01 | 0.23 | 0.25 | 0.15 | 0.25 | 1.00 |  |  |  |  |  |  |
| Educational games | 0.01 | -0.02 | -0.01 | 0.15 | 0.14 | 0.09 | 0.06 | -0.05 | -0.04 | -0.03 | -0.04 | -0.03 | 1.00 |  |  |  |  |  |
| Role-playing games | 0.02 | 0.01 | 0.00 | 0.10 | 0.07 | 0.04 | 0.05 | -0.00 | 0.05 | 0.03 | 0.02 | 0.01 | 0.23 | 1.00 |  |  |  |  |
| Moving activities | 0.01 | 0.06 | 0.06 | 0.11 | 0.11 | 0.08 | 0.03 | 0.03 | 0.04 | -0.02 | 0.04 | 0.07 | 0.23 | 0.18 | 1.00 |  |  |  |
| Total screen time | -0.07 | 0.15 | 0.17 | -0.12 | -0.07 | 0.02 | 0.01 | 0.14 | 0.14 | 0.09 | 0.17 | 0.16 | -0.04 | 0.00 | 0.04 | 1.00 |  |  |
| Total sleep duration | -0.08 | 0.02 | -0.01 | 0.00 | 0.03 | 0.00 | 0.06 | 0.00 | -0.02 | -0.00 | 0.00 | 0.01 | -0.02 | 0.04 | 0.04 | -0.01 | 1.00 |  |
| Trouble falling asleep & night-time awakenings | 0.10 | 0.02 | 0.06 | -0.03 | -0.03 | 0.01 | -0.05 | 0.06 | 0.07 | 0.04 | 0.05 | 0.04 | -0.00 | 0.01 | 0.01 | 0.05 | -0.23 | 1.00 |

* Pearson correlation coefficients.

***Supplementary Table 1.*** *Missing data in the sample for the lifestyle patterns analysis. The ELFE study (N=13,121).*

|  | **Missing data**  **n (%)** |
| --- | --- |
|  |  |
| Breastfeeding duration | 146 (1.0) |
| Introduction of complementary feeding before age 4 months | 3,153 (24.0) |
| Introduction of sweet beverages before age 10 months | 3,484 (26.6) |
| Vegetables | 86 (0.7) |
| Fruits | 87 (0.7) |
| Fish | 90 (0.7) |
| Dairy products | 84 (0.6) |
| Discretionary savory foods | 90 (0.7) |
| Discretionary sweet foods | 89 (0.7) |
| Processed meat | 90 (0.7) |
| French fries | 89 (0.7) |
| Sweet beverages | 83 (0.6) |
| Educational games | 0 (0) |
| Role-playing games | 0 (0) |
| Moving activities | 0 (0) |
| Total screen time | 1,003 (7.6) |
| Total sleep duration | 526 (4.0) |
| Trouble falling asleep & night-time awakenings | 211 (1.6) |

***Supplementary Table 3.*** *Proportion of missing data and imputation models used for the analysis of overweight/obesity risk at age 5 years. The ELFE study (n=8,388).*

| **Variables** | **Type** | **Imputation model** | **% missing** |
| --- | --- | --- | --- |
| **Exposures^a^** |  |  |  |
| 3 lifestyle patterns (PCA scores)^b^ | Continuous | Predictive mean matching | 7 |
| **Covariate^a^** |  |  |  |
| Parity | Dichotomous | Logistic | 1.2 |
| Maternal age at delivery | Continuous | Predictive mean matching | 0.4 |
| Child’s sex | Dichotomous | No missing data | 0.0 |
| Maternal BMI | Continuous | Predictive mean matching | 1.3 |
| Paternal BMI | Continuous | Predictive mean matching | 15.0 |
| Maternal smoking during pregnancy | Dichotomous | Logistic | 0.9 |
| Paternal smoking | Dichotomous | Logistic | 15.0 |
| Caesarean delivery | Dichotomous | Logistic | 2.7 |
| Birth weight | Continuous | Predictive mean matching | 2.8 |
| Parents’ migration status | Categorical | Multinomial | 11.7 |
| Parents’ education level | Categorical | Multinomial | 9.4 |
| Household income per consumption unit | Categorical | Multinomial | 5.3 |

^a^ In addition to the variables in the table, the following variables were used as predictors in the imputation model (except lifestyle patterns, which were not used as predictors): mother’s and father’s country of birth, mother's marital/cohabitation status, maternal and paternal occupational category, paternal age at delivery. Overweight/obesity risk at age 5 years was not used as a predictor. ^b^ “Early complementary feeding, discretionary consumption, high screen time”, “Balanced diet, non-screen leisure activities”, and “Healthy feeding practices, low dairy consumption, suboptimal sleep” patterns.

PCA: principal component analysis

***Supplementary Table 4.*** *Proportion of missing data and imputation models used for analysis of age at adiposity rebound. The ELFE study (n=7,770)*

| **Variables** | **Type** | **Imputation model** | **% missing** |
| --- | --- | --- | --- |
| **Exposures^a^** |  |  |  |
| 3 lifestyle patterns (PCA scores)^b^ | Continuous | Predictive mean matching | 2.7 |
| **Covariate^a^** |  |  |  |
| Parity | Dichotomous | Logistic | 1.2 |
| Maternal age at delivery | Continuous | Predictive mean matching | 0.4 |
| Child’s sex | Dichotomous | No missing data | 0.0 |
| Maternal BMI | Continuous | Predictive mean matching | 1.2 |
| Paternal BMI | Continuous | Predictive mean matching | 10.7 |
| Maternal smoking during pregnancy | Dichotomous | Logistic | 0.9 |
| Paternal smoking | Dichotomous | Logistic | 10.6 |
| Caesarean delivery | Dichotomous | Logistic | 2.5 |
| Birth weight | Continuous | Predictive mean matching | 2.6 |
| Parents’ migration status | Categorical | Multinomial | 8.3 |
| Parents’ education level | Categorical | Multinomial | 6.3 |
| Household income per consumption unit | Categorical | Multinomial | 3.8 |

^a^ In addition to the variables in the table, the following variables were used as predictors in the imputation model (except lifestyle patterns, which were not used as predictors): mother’s and father’s country of birth, mother's marital/cohabitation status, maternal and paternal occupational category, paternal age at delivery. Age at adiposity rebound was not used as a predictor. ^b^ “Early complementary feeding, discretionary consumption, high screen time”, “Balanced diet, non-screen leisure activities”, and “Healthy feeding practices, low dairy consumption, suboptimal sleep” patterns.

PCA: principal component analysis

***Supplementary Table 5.*** *Comparison of included and excluded children for the overweight/obesity risk analysis. The ELFE study.*

|  | **Included  N = 8,388** | **Excluded**  **N = 9,941** | **p-value**^a^ |
| --- | --- | --- | --- |
| **Maternal age at delivery**^b^ (years) | 31.1 (28.2 – 34.4) | 29.9 (26.1 – 33.7) | <0.001 |
| **Single motherhood** |  |  | <0.001 |
| Yes | 2.0% (159) | 6.3% (524) |  |
| **Parity (first child)** |  |  | 0.006 |
| Yes | 47% (3,900) | 45% (4,348) |  |
| **Parents’ migration status** |  |  | <0.001 |
| Non-immigrant | 72.0% (5,333) | 61.7% (3,867) |  |
| At least one parent descendant of immigrant | 17.5% (1,295) | 17.9% (1,121) |  |
| At least one parent immigrant | 10.5% (778) | 20.5% (1,284) |  |
| **Parents’ education level** |  |  | <0.001 |
| ≥ 3-year university | 54.8% (4,488) | 35.3% (2,907) |  |
| 1- to 2-year university | 22.6% (1,846) | 19.8% (1,631) |  |
| ≤ High school degree | 22.6% (1,852) | 44.9% (3,701) |  |
| **Household income per consumption unit**^b^ (€/month) | 1,667 (1,250-2,082) | 1,389 (958-1,778) | <0.001 |
| **Sex of the child** |  |  | 0.95 |
| Girl | 48.6% (4,079) | 48.6% (4 780) |  |

^a^ Wilcoxon rank sum test for numeric data; chi-squared test for categorical data

^b^ Median (IQR)

***Supplementary Table 6.*** *Comparison of included and excluded children for the analysis of age at adiposity rebound. The ELFE study.*

|  | **Included  N = 7,770** | **Excluded**  **N = 10,559** | **p-value**^a^ |
| --- | --- | --- | --- |
| **Maternal age at delivery**^b^ (years) | 31.3 (28.6–34.5) | 29.7 (26.0–33.6) | <0.001 |
| **Single motherhood** |  |  | <0.001 |
| Yes | 1.4% (110) | 6.5% (573) |  |
| **Parity (first child)** |  |  | <0.001 |
| Yes | 47.8% (3,669) | 44.5% (4,579) |  |
| **Parents’ migration status** |  |  | <0.001 |
| Non-immigrant | 74.9% (5,335) | 59.0% (3,865) |  |
| At least one parent descendant of immigrant | 16.9% (1,207) | 18.5% (1,209) |  |
| At least one parent immigrant | 8.2% (584) | 22.6% (1,478) |  |
| **Parents’ education level** |  |  | <0.001 |
| ≥ 3-year university | 59.6% (4,569) | 32.3% (2,826) |  |
| 1- to 2-year university | 22.5% (1,724) | 20.0% (1,753) |  |
| ≤ High school degree | 17.9% (1,371) | 47.7% (4,182) |  |
| **Household income per consumption unit**^b^ (€/month) | 1,667 (1,333-2,143) | 1,333 (952-1,714) | <0.001 |
| **Sex of the child** |  |  | 0.872 |
| Girl | 48.5% (3,770) | 48.7% (5,089) |  |

^a^ Wilcoxon rank sum test for numeric data; chi-squared test for categorical data

^b^ Median (IQR)

***Supplementary Table 7.*** *Principal component analysis loadings for lifestyle patterns (LPs). The ELFE study with complete cases (N=8,675).*

|  | **PCA Loadings** | | |
| --- | --- | --- | --- |
|  | **LP1**^a^ | **LP2**^b^ | **LP3**^c^ |
| Breastfeeding duration (months) | -0.13 | 0.13 | **0.58** |
| Introduction of complementary feeding before age 4 months (yes) | **0.39** | -0.09 | **-0.33** |
| Introduction of sweet beverages before age 10 months (yes) | **0.45** | -0.03 | -0.11 |
| Vegetables (/day) | -0.19 | **0.62** | 0.18 |
| Fruits (/day) | -0.05 | **0.62** | 0.11 |
| Fish (/day) | 0.13 | **0.39** | 0.09 |
| Dairy products (/day) | 0.06 | 0.23 | **-0.35** |
| Discretionary savoury foods (/day) | **0.60** | 0.06 | 0.16 |
| Discretionary sweet foods (/day) | **0.54** | 0.06 | 0.06 |
| Processed meat (/day) | **0.44** | 0.06 | 0.10 |
| French fries (/day) | **0.63** | 0.07 | 0.11 |
| Sweet beverages (/day) | **0.59** | 0.09 | 0.04 |
| Educational games (/week) | -0.10 | **0.55** | -0.09 |
| Pretend plays (/week) | 0.03 | **0.47** | -0.16 |
| Movement activities (/week) | 0.12 | **0.49** | -0.12 |
| Total screen time (hours/day) | **0.46** | -0.11 | -0.11 |
| Total sleep duration (hours/day) | -0.02 | 0.09 | **-0.56** |
| Trouble falling asleep & night-time awakenings (/week) | 0.15 | -0.07 | **0.56** |
| % variance explained | 12.7 | 10.0 | 7.6 |

^a^ LP1: “Early complementary feeding, discretionary consumption, high screen time”; ^b^ LP2: “Balanced diet, non-screen leisure activities”; ^c^ LP3: “Healthy feeding practices, low dairy consumption, suboptimal sleep”. In bold, absolute value of factor loadings ≥ 0.30.

***Supplementary Table 8.*** *Associations between lifestyle patterns (LPs) and anthropometric outcomes*. The ELFE study with complete cases.*

|  | **Overweight/obesity at age 5**  N=6,224 | |  | **Age at adiposity rebound**  N=6,230 | |
| --- | --- | --- | --- | --- | --- |
|  | **OR [95% CI]** | **p-value** |  | **β [95% CI]** | **p-value** |
| **LP1**^a^ | 1.11 [1.04, 1.19] | 0.003 |  | -10.1 [-18.2, -2.0] | <0.001 |
| **LP2**^b^ | 0.98 [0.91, 1.06] | 0.649 |  | 0.1 [-7.6, 7.7] | 0.477 |
| **LP3**^c^ | 1.06 [0.97, 1.16] | 0.194 |  | -3.7 [-12.5, 5.1] | 0.231 |

*Logistic regression model for overweight/obesity and linear regression model for age at adiposity rebound. These two multivariable models included the three LPs and were adjusted for child sex, maternal age at delivery, parity, maternal and paternal BMI, maternal smoking during pregnancy, paternal smoking, caesarean delivery, birth weight, household income, parents’ migration status and parents’ education level.

^a^ LP1: “Early complementary feeding, discretionary consumption, high screen time”; ^b^ LP2: “Balanced diet, non-screen leisure activities”; ^c^ LP3: “Healthy feeding practices, low dairy consumption, suboptimal sleep”.
