# Supplementary material for: Association of early childhood lifestyle patterns with overweight in preschoolers and timing of adiposity rebound
Source: Int J Behav Nutr Phys Act. 2026 Jun 25;23:86. doi: 10.1186/s12966-026-01941-w (PMC13587429; doi:10.1186/s12966-026-01941-w)
Supplement: Supplementary file 2 [file 12966_2026_1941_MOESM2_ESM.docx]

STROBE Statement—Checklist of items that should be included in reports of ***cohort studies***

|  | Item No | Recommendation | Respected? | Comments and quotes |
| --- | --- | --- | --- | --- |
| **Title and abstract** | 1 | (*a*) Indicate the study’s design with a commonly used term in the title or the abstract | Yes | The abstract provides a clear indication of the study design. Quote : “the French Etude Longitudinale Française depuis l'Enfance (ELFE) birth cohort …” |
|  |  | (*b*) Provide in the abstract an informative and balanced summary of what was done and what was found | Yes | The abstract provides a balanced summary of the study’s methodology, results, and conclusions. Quote: “Associations with overweight/obesity risk at 5 years (IOTF definition; n=8 388)” |
| Introduction | | |  |  |
| Background/rationale | 2 | Explain the scientific background and rationale for the investigation being reported | Yes | Page 4, Lines 2-24 and Page 5, Lines 1-5 |
| Objectives | 3 | State specific objectives, including any prespecified hypotheses | Yes | Page 5, Lines 6-9 |
| Methods | | |  |  |
| Study design | 4 | Present key elements of study design early in the paper | Yes | Page 5, Lines 12-13 |
| Setting | 5 | Describe the setting, locations, and relevant dates, including periods of recruitment, exposure, follow-up, and data collection | Yes | Page 5, Lines 13-22 |
| Participants | 6 | (*a*) Give the eligibility criteria, and the sources and methods of selection of participants. Describe methods of follow-up | Yes | Page 5, Lines 13-22 |
|  |  | (*b*) For matched studies, give matching criteria and number of exposed and unexposed | N/A |  |
| Variables | 7 | Clearly define all outcomes, exposures, predictors, potential confounders, and effect modifiers. Give diagnostic criteria, if applicable | Yes | Page 6, Lines 1-24, Pages 7, Lines 1-24 and Page 8, Lines 1-20 |
| Data sources/ measurement | 8* | For each variable of interest, give sources of data and details of methods of assessment (measurement). Describe comparability of assessment methods if there is more than one group | Yes | Page 6, Lines 1-24, Pages 7, Lines 1-24 and Page 8, Lines 1-20, Supplementary Table 1 |
| Bias | 9 | Describe any efforts to address potential sources of bias | Yes | Imputation with regularized interactive PCA: Page 9, Lines 12-14, Supplementary Table 2,  Multivariate Imputation by Chained Equations: Page 10, Lines 1-12, Supplementary Table 3, Supplementary Table 4  Adjustments: Page 10, Lines 15-16  Attrition bias (complete cases): Page 11, Lines 23-24 |
| Study size | 10 | Explain how the study size was arrived at | N/A |  |
| Quantitative variables | 11 | Explain how quantitative variables were handled in the analyses. If applicable, describe which groupings were chosen and why | Yes | Page 9, Lines 7-24, Page 10, Lines 1-20 |
| Statistical methods | 12 | (*a*) Describe all statistical methods, including those used to control for confounding | Yes | Page 9, Lines 7-24, Page 10, Lines 1-20 |
|  |  | (*b*) Describe any methods used to examine subgroups and interactions | N/A |  |
|  |  | (*c*) Explain how missing data were addressed | Yes | Page 9, Lines 12-14, Page 10, Lines 1-12, Supplementary Tables 2,3,4 |
|  |  | (*d*) If applicable, explain how loss to follow-up was addressed | N/A |  |
|  |  | (*e*) Describe any sensitivity analyses | Yes | ¨Page 10, Lines 17-18 |
| Results | | |  |  |
| Participants | 13* | (a) Report numbers of individuals at each stage of study—eg numbers potentially eligible, examined for eligibility, confirmed eligible, included in the study, completing follow-up, and analysed | Yes | Page 8, Lines 22-24, Page 9, Lines 1-3 |
|  |  | (b) Give reasons for non-participation at each stage | Yes | Flow chart in Figure 1 |
|  |  | (c) Consider use of a flow diagram | Yes | Flow chart in Figure 1 |
| Descriptive data | 14* | (a) Give characteristics of study participants (eg demographic, clinical, social) and information on exposures and potential confounders | Yes | Table 1, Table 2, Supplementary Table 5, Supplementary Table 6 |
|  |  | (b) Indicate number of participants with missing data for each variable of interest | Yes | Supplementary Table 2, Supplementary Table 3, Supplementary Table 4 |
|  |  | (c) Summarise follow-up time (eg, average and total amount) | Yes | Page 8, Lines 22-24, Page 9, Lines 1-3 |
| Outcome data | 15* | Report numbers of outcome events or summary measures over time | Yes | Page 11, Lines 3-6 |
| Main results | 16 | (*a*) Give unadjusted estimates and, if applicable, confounder-adjusted estimates and their precision (eg, 95% confidence interval). Make clear which confounders were adjusted for and why they were included | Yes | Page 11, Lines 19-24, Page 12, Lines 1-4, Table 3 |
|  |  | (*b*) Report category boundaries when continuous variables were categorized | N/A |  |
|  |  | (*c*) If relevant, consider translating estimates of relative risk into absolute risk for a meaningful time period | N/A |  |
| Other analyses | 17 | Report other analyses done—eg analyses of subgroups and interactions, and sensitivity analyses | Yes | Supplementary Table 7, Supplementary Table 8 |
| Discussion | | |  |  |
| Key results | 18 | Summarise key results with reference to study objectives | Yes | Page 12, Lines 10-17 |
| Limitations | 19 | Discuss limitations of the study, taking into account sources of potential bias or imprecision. Discuss both direction and magnitude of any potential bias | Yes | Page 17, Lines 1-24 |
| Interpretation | 20 | Give a cautious overall interpretation of results considering objectives, limitations, multiplicity of analyses, results from similar studies, and other relevant evidence | Yes | Page 12, Lines 19-23, Page 13, Lines 1-25, Page 14, Lines 1-24, Page 15, Lines 1-23, Page 16, Lines 1-23 |
| Generalisability | 21 | Discuss the generalisability (external validity) of the study results | N/A |  |
| Other information | | |  |  |
| Funding | 22 | Give the source of funding and the role of the funders for the present study and, if applicable, for the original study on which the present article is based | Yes | Page 19, Lines 22, Page 20, Lines 1-17 |

*Give information separately for exposed and unexposed groups.

**Note:** An Explanation and Elaboration article discusses each checklist item and gives methodological background and published examples of transparent reporting. The STROBE checklist is best used in conjunction with this article (freely available on the Web sites of PLoS Medicine at http://www.plosmedicine.org/, Annals of Internal Medicine at http://www.annals.org/, and Epidemiology at http://www.epidem.com/). Information on the STROBE Initiative is available at http://www.strobe-statement.org.
